# Preventive effect of sensorimotor exercise and resistance training on chemotherapy-induced peripheral neuropathy: a randomised-controlled trial

**DOI:** 10.1038/s41416-021-01471-1

**Published:** 2021-07-05

**Authors:** Jana Müller, Markus Weiler, Andreas Schneeweiss, Georg Martin Haag, Karen Steindorf, Wolfgang Wick, Joachim Wiskemann

**Affiliations:** 1 Working Group Exercise Oncology, Division of Medical Oncology, National Center for Tumor Diseases (NCT) and Heidelberg University Hospital, Heidelberg, Germany; 2 German Cancer Research Center (DKFZ), Heidelberg, Germany; 3 Institute of Sports and Sport Science, Heidelberg University, Heidelberg, Germany; 4 Department of Neurology, Heidelberg University Hospital, Heidelberg, Germany; 5 Division of Gynecological Oncology, National Center for Tumor Diseases (NCT) and Heidelberg University Hospital, Heidelberg, Germany; 6 Division of Medical Oncology, National Center for Tumor Diseases (NCT) and Heidelberg University Hospital, Heidelberg, Germany; 7 Division of Physical Activity, Prevention and Cancer, German Cancer Research Center (DKFZ) and National Center for Tumor Diseases (NCT), Heidelberg, Germany

**Keywords:** Neurological manifestations, Breast cancer, Randomized controlled trials, Quality of life, Lifestyle modification

## Abstract

**Background:**

Chemotherapy-induced peripheral neuropathy (CIPN) is a common, unpleasant and usually long-lasting side effect of neurotoxic chemotherapeutic agents. This study aimed to investigate the preventive potential of sensorimotor- (SMT) and resistance training (RT) on CIPN.

**Methods:**

Patients (*N* = 170) were randomised to SMT, RT or usual care (UC). Both exercise groups trained 3×/week for a total of 105 min/week during neurotoxic chemotherapy (mean length: 20 weeks). Before and 3 weeks after neurotoxic chemotherapy, CIPN signs/symptoms were assessed via Total Neuropathy Score (TNSr; primary endpoint) and EORTC QLQ-CIPN15 questionnaire. In addition, balance (centre of pressure), muscle strength (isokinetic), quality of life (QoL, EORTC QLQ-C30) and relative chemotherapy dose intensity (RDI) were investigated. The follow-up period covered 6 months after the end of chemotherapy.

**Results:**

Intention-to-treat analyses (*N* = 159) revealed no differences regarding CIPN signs/symptoms. Exploratory per-protocol analyses (minimum training attendance rate 67%; *N* = 89) indicated that subjectively perceived sensory symptoms in the feet increased less during chemotherapy in the adherent exercisers (pooled group: SMT+RT) than in the UC group (−8.3 points (−16.1 to −0.4); *P* = 0.039, ES = 1.27). Furthermore, adherent exercisers received a higher RDI (96.6 ± 4.8 vs. 92.2 ± 9.4; *P* = 0.045), showed a better course of muscular strength (+20.8 Nm (11.2–30.4); *P* < 0.001, ES = 0.57) and QoL (+12.9 points (3.9–21.8); *P* = 0.005, ES = 0.64). During follow-up, CIPN signs/symptoms persisted in all groups.

**Conclusions:**

This study demonstrates that SMT and/or RT alleviate subjectively perceived sensory CIPN symptoms in the feet and other clinically relevant cancer therapy-related outcomes, if an appropriate training stimulus is achieved.

**Clinical trial registration:**

NCT02871284.

## Introduction

Tingling, burning, numbness and pain in hands and/or feet may be observed from the first administration of neurotoxic drugs such as taxanes, platinum compounds or vinca alkaloids [1, 2]. The severity and persistence of chemotherapy-induced peripheral neuropathy (CIPN) are mainly dependent on drug type and cumulative dose, but probably also on comorbidities and lifestyle factors such as obesity and low moderate-to-vigorous physical activity [3, 4]. The primary sensory symptoms and resulting functional limitations, such as balance and gait difficulties, may persist over several years/decades in up to 50% of patients [5–8], causing reduced individual independence and quality of life [6], but also probably increased cancer recurrence and mortality rates, due to chemotherapy dose reductions and early treatment termination [9].

The reduction of chemotherapy dose is currently the only way to prevent the progression of CIPN symptoms. However, the body of research is constantly growing investigating the effects of various prevention and rehabilitation measures, such as exercise therapy. After chemotherapy, exercise is shown to positively affect various aspects of CIPN [10–15]. However, the preventive potential has been so far less investigated and the results are sometimes divergent. Positive intervention effects were found for deep sensitivity [16], perception of hot and coldness [17] and static balance performance [18]. All other studies were not able to detect a positive influence on CIPN signs/symptoms or functional limitations [19–21], which might be due to the following methodological issues: small sample sizes (*N* = 19–43) [16, 18–21], blurred baseline values by performing baseline measurement after the first chemotherapy administration [16, 21], and rudimentary CIPN assessment. Regarding CIPN assessment, it is recommended in the current literature to combine both subjective and objective CIPN diagnostics [22]. However, only one study complies with the current literature recommendations [19]. All other studies used either singular subjective assessments (ranging from simple, non-psychometrically tested symptom queries to the use of recommended questionnaires [17, 18, 20, 21]), or a singular objective test (tuning fork [16]). On this basis, we conducted a single-centre randomised-controlled three-arm intervention trial. The primary aim of the PIC study was to evaluate the preventive potential of sensorimotor exercise training (SMT) and resistance training (RT) versus usual care (UC) during neurotoxic chemotherapy on clinically objectified CIPN signs/symptoms by means of the Total Neuropathy Score (TNSr). The SMT represented a specific training approach guided by the CIPN symptoms (especially the balance impairment), while the RT was tested as a non-specific training approach. We hypothesised that patients randomised to the SMT or RT group would have a smaller change on the TNSr score over the course of neurotoxic chemotherapy in comparison to patients receiving UC.

## Methods

### Study design and participants

The PIC study was a single-centre randomised-controlled three-arm exercise intervention trial. Ethical approval was obtained (Ethics Committee Medical Faculty University of Heidelberg: S-630/2015) and the trial was registered before activation (ClinicalTrials.gov: NCT02871284). Patients were eligible if they were ≥ 18 years of age and were admitted to receive neurotoxic chemotherapy, which had not been started at the time of study assignment and baseline testing (see Table 1 for complete inclusion/exclusion criteria).

**Table 1:** Study inclusion and exclusion criteria.

Inclusion criteria	• Age ≥18 years
	• Diagnosed with cancer and assigned to receive a chemotherapeutic regimen containing at least one of the following agents: - a platinum analogue, e.g. cisplatin, carboplatin, oxaliplatin - a vinca alkaloid, e.g. vincristine - a taxane, e.g. paclitaxel, docetaxel - suramin - thalidomide or lenalidomide - bortezomib
	• Physical capability to follow the training programme implemented within the exercise intervention groups
Exclusion criteria	• Known peripheral neuropathy of any kind or any peripheral neuropathic signs or symptoms at baseline
	• Positive family history for any hereditary peripheral neuropathy
	• Known metastasis to the central or peripheral nervous system
	• Any physical or mental handicap that would hamper the performance of the training programme implemented within the exercise intervention groups
	• Known history of alcohol or illegal drug abuse or any constellation of lab values suggesting alcoholism, e.g. elevated GGT, MCV, CDT

### Procedures

**Fig. 1:**
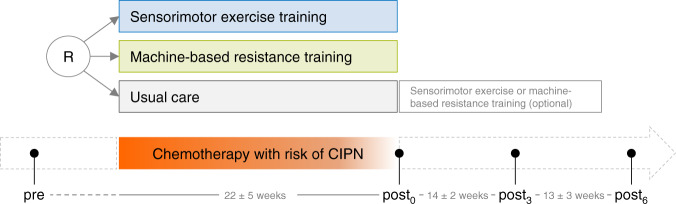
Study design. *CIPN* chemotherapy-induced peripheral neuropathy, *pre* assessment point before neurotoxic chemotherapy, *post*_*0*_ assessment point 3 weeks after neurotoxic chemotherapy, *post*_*3*_ assessment point 3 months after post_0_, *post*_*6*_ assessment point 6 months after post_0_, *R* randomisation.

Potentially eligible patients were identified by their physicians or through hospital records at the National Center for Tumor Diseases (NCT, Heidelberg, Germany) or regional cooperation clinics between March 2016 and June 2018. After providing written informed consent and completed baseline testing (pre), patients were randomly assigned to an exercise intervention (SMT or RT) or UC group. The allocation was done by an independent person (i.e. not involved in patient recruitment) based on allocation lists generated by a computerised random number generator. These lists were generated prior to the inclusion of the first patient and were based on blocked randomisation with random block sizes of three and six, stratified by gender and type of treatment (taxanes, platinum derivatives, vinca alkaloids, combined neurotoxic chemotherapy). The personnel involved in the recruitment and baseline assessments were not involved in this process and had no access to these lists throughout the whole study. Three weeks after completion of the individual chemotherapy regime post_0_ assessment took place. Follow-up assessments were scheduled 3 (post_3_) and 6 months (post_6_) after post_0_ (Fig. 1). All assessments were carried out in accordance with The Code of Ethics of the World Medical Association (Declaration of Helsinki, 2013).

### Outcomes

#### CIPN signs and symptoms

The primary endpoint was the Total Neuropathy Score in its reduced version (TNSr) [23]. The TNSr represents a sum score of patient-reported symptoms (sensory, motor, autonomic) and clinical examinations of CIPN signs/symptoms (pinprick, reflexes, deep sensitivity, strength) as well as nerve conduction studies (NCS; motor: compound muscle action potential amplitude of peroneal nerve (CMAP), sensory: sensory nerve action potential amplitude of sural nerve (SNAP)), with higher values reflecting a greater symptom burden. In addition, the TNSc (TNSr without NCS), TNSm (without NCS and autonomic symptoms [24]) and the result variables of the NCS are reported separately: CMAP, SNAP, and nerve conduction velocities (NCV). To ensure data quality, all TNS assessments were blinded and performed according to general standards [23]. In addition, 94% of the NCS (534 of 568 measurements) were carried out by the same technician with longstanding experience in clinical neurophysiology and peripheral neuropathy.

Furthermore, CIPN symptoms were assessed based on patients’ perception by using the CIPN questionnaire of the European Organization for Research and Treatment of Cancer (EORTC QLQ-CIPN15) [25]. In contrast to the initially published scoring manual, the mean sum score was calculated over 15 instead of 20 items (hereinafter referred to as CIPN-15), with higher values expressing higher CIPN symptoms (score range 0–100) [26]. Since our main exercise intervention (SMT) particularly focused on the lower extremities, we exploratory defined two separate scores for sensory (items 2, 4, 6, 9) and motor symptoms (items 8, 14, 15) in the feet, in accordance to the lower extremity score [27].

#### Functional assessments

Postural control was assessed with a force plate (AMTI, AccuSway optimised, Watertown, USA). The detailed testing procedure is described elsewhere [28]. Briefly, patients were asked to stand as still as possible in bipedal stance with eyes closed (BP_EC_) for 30 s. The best trial out of two was reported (lowest centre of pressure (COP) value for total mean velocity). In addition, we determined the average time of two trials patients were able to stand on one leg with open eyes (MP_EO_). Maximal voluntary isometric contraction for quadriceps was measured with an isokinetic dynamometer (IsoMed 2000-system B-series version, D&R Ferstl GmbH, Hemau, Germany). The test setup included a maximum force generation against the dynamometer arm for 6 s at a knee angle of 36°. Maximal peak torque was measured in the dominant leg, which was defined based on the higher peak torque of the right and left leg at baseline.

#### Patient-reported outcomes

Quality of life (QoL) was assessed with the validated EORTC QLQ-C30 questionnaire (version 3.0) [29, 30]. Fear of falling was assessed via Fall Efficacy Scale (FES-I) [31]. In addition, the number of falls was assessed (a) at baseline (pre), referring to the last 6 months, and (b) weekly during chemotherapy via telephone calls.

Demographic, clinical and behavioural data (including minutes of exercise per week [28]) were collected from medical records and study-specific forms. Relative dose intensity (RDI) and relative cumulative dose were calculated according to guidelines [32]. Concomitant CIPN prevention and treatment measures (e.g. cryotherapy, duloxetine intake) were queried from the patients.

#### Exercise adherence and tolerability

Based on training documents completed by the patients, adherence data were evaluated [33]. The reasons for missed training sessions and training-related adverse events were queried in weekly telephone calls.

### Exercise interventions

#### Sensorimotor exercise training

The SMT was scheduled 3×/week for 35 min each. During an introductory one-to-one training session, the patients received a catalogue of exercises, including 45 illustrated exercise cards, and necessary training materials (e.g. Airex balance pad). The patients exercised either at home or in an open supervised training session at the NCT. Each exercise was carried out 3 × 30 s with at least 30 s pause between sets. Patients were asked to progress their training based on individually perceived difficulty. Figure S1 and Table S1a provide further details.

#### Resistance training

The RT included a machine-based RT 2×/week for 45 min each, and a 15 min home-based training once a week. The detailed training descriptions can be found in Table S1b. Briefly, the machine-based RT consisted of a maximum of eight exercises per session and was performed in an experienced exercise oncology training facility (OnkoAktiv Network). After two familiarisation sessions, a one-repetition-maximum strength test (1RM) was conducted at each resistance machine. Its results were used to define initial training weights based on current guidelines (70–80% 1RM) [34]. The home-based RT consisted of progressively designed core stability exercises.

#### Usual care

The control group received usual care (UC) without additional information about physical activity. During follow-up (post_0_–post_6_), UC patients had the opportunity to participate in one of the interventions described above for a maximum of 12 weeks.

All patients received weekly phone calls to monitor nutritional status (to identify the risk of malnutrition at an early stage) and fall history as well as training compliance and potential adverse events related to the intervention programme, if applicable.

### Statistical analysis

The sample-size estimation was based on the main outcome criterion, the change of the TNSr from pre to post_0_. Sample-size calculation was performed by Monte-Carlo simulations of the power for the Kruskal-Wallis Test. Simulations were performed with the following input parameters: (i) equal allocation between the three groups, (ii) equidistant population means, (iii) normalised equal distribution, (iv) α = 5%. Under these assumptions, a sample size of 246 (82 per group) was calculated to achieve a power of 80%. Assuming a maximal drop-out rate of 20%, it was planned to recruit 300 patients.

Baseline differences were tested by Kruskal-Wallis or Chi^2^/Fisher’s exact test in the case of categorical variables. The primary analyses followed an intention-to-treat (ITT) approach. Secondary analyses included a per-protocol (PP) approach where patients with an attendance rate of lower than 66.67% of planned training sessions were excluded from analyses [35]. In addition, a second exploratory PP_EX_ analyses with both exercise groups combined (only adherent exercisers; EX) vs. UC was conducted. The combination of the two intervention groups was substantiated due to comparable neuromuscular training adaptations [36]. Analysis of covariance (ANCOVA) was used to test (i) intervention effects (pre–post_0_), and (ii) changes during follow-up (post_0_–post_3_, post_0_–post_6_) with the change scores of the respective comparison being the dependent variable, the intervention groups (SMT vs. RT vs. UC; EX vs. UC) as independent variable and stratification variables (gender and treatment), age, and baseline (pre–post_0_) or post_0_ measure (post_0_–post_3_, post_0_–post_6_) as covariates. ANCOVA was based on complete case analyses per analysed study period (pre–post_0_, post_0_–post_3_, post_0_–post_6_). The analysed sample size is indicated per variable and analysis. No adjustments for multiple comparisons for the follow-up comparisons and secondary outcomes and analyses were made, as these were considered to be explorative. Standardised effect sizes (ES) were calculated for within-group and between-group comparisons for all outcomes by respectively dividing the adjusted mean change or the adjusted between-group difference by the baseline standard deviation. For ease of presentation of the between-group comparisons, ES received a positive sign if it was in favour of the first group of the following comparisons: SMT vs. UC, RT vs. UC, SMT vs. RT, and EX vs. UC. All statistical tests were two-sided, and *P* < 0.05 was considered statistically significant. SAS Enterprise Guide 7.1 (SAS Institute Inc., USA) was used for all analyses.

## Results

**Fig. 2:**
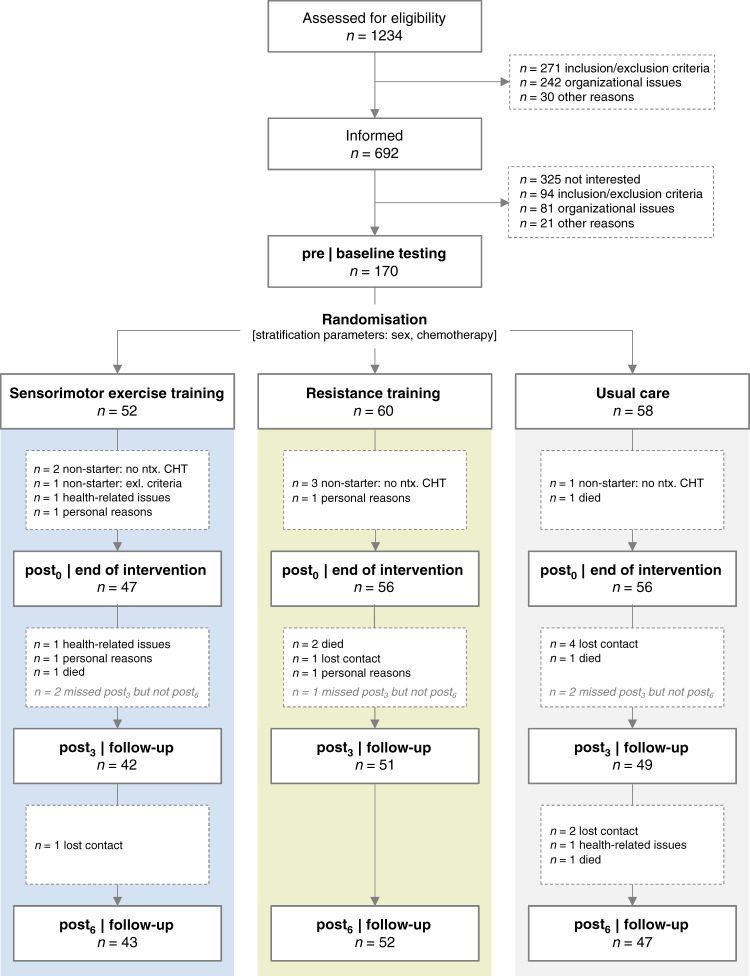
CONSORT flow chart. *Pre* assessment point before neurotoxic chemotherapy, *post*_*0*_ assessment point 3 weeks after neurotoxic chemotherapy, *post*_*3*_ assessment point three months after post_0_, *post*_*6*_ assessment point six months after post_0_.

One hundred and seventy patients (mean age 53.3 years) were randomised after baseline testing, of which *N* = 159 completed the intervention period and were included in the ITT analysis (Fig. 2). Most patients were female (85%) and had breast cancer (74%) (Table 2 and Table S2). Due to a poor recruitment rate (25%), we were unable to achieve our intended sample size within the given project time.

**Table 2:** Patient characteristics.

	Total	SMT	RT	UC	*P* value	
Demographic profile						
Number of patients (*n*)	163 (100%)	49 (30%)	57 (35%)	57 (35%)	–	
Number of female patients (*n*)	138 (85%)	41 (84%)	48 (84%)	49 (86%)	0.942	
Age (years, mean ± SD)	53.3 ± 11.5	51.7 ± 10.8	53.4 ± 11.7	54.5 ± 11.9	0.396	
Married (*n* (%))	124 (78%)	38 (78%)	43 (78%)	43 (78%)	0.996	
University degree (*n* (%))	54 (34%)	22 (45%)	15 (27%)	17 (31%)	0.140	
Medical profile						
Height (cm, mean ± SD)	167.3 ± 6.8	168.5 ± 7.5	167.5 ± 6.7	166 ± 6	0.241	
Weight (kg, mean ± SD)	72.9 ± 14.3	74.2 ± 15.7	74.8 ± 13.3	70 ± 13.7	0.108	
BMI (kg/m^2^, mean ± SD)	26.1 ± 5	26.2 ± 5.6	26.7 ± 4.7	25.4 ± 4.8	0.272	
Comorbidities (*n* (%))						
None	21 (13%)	9 (18%)	4 (7%)	8 (14%)	0.209	
Cardiovascular	60 (37%)	19 (39%)	20 (35%)	21 (37%)	0.926	
Musculoskeletal	94 (58%)	22 (45%)	40 (70%)	32 (56%)	**0.030**	
Neurological	14 (9%)	3 (6%)	7 (12%)	4 (7%)	0.537	
Endocrine/metabolic	21 (13%)	7 (14%)	10 (18%)	4 (7%)	0.230	
[diabetes]	7 (4%)	2 (4%)	3 (5%)	2 (4%)	1.00	
Psychiatric	9 (6%)	3 (6%)	3 (5%)	3 (5%)	1.00	
Prior cancer diagnosis^a^	20 (12%)	7 (14%)	3 (5%)	10 (18%)	0.119	
Oncological diagnosis (*n* (%))						
Breast cancer	121 (74%)	36 (73%)	41 (72%)	44 (77%)	0.805^b^	
Pancreatic cancer	9 (6%)	2 (4%)	3 (5%)	4 (7%)		
Prostate cancer	5 (3%)	2 (4%)		3 (5%)		
Stomach cancer	5 (3%)	2 (4%)	2 (4%)	1 (2%)		
Oesophagus cancer	4 (2%)	1 (2%)	1 (2%)	2 (4%)		
Colon cancer	4 (2%)	3 (6%)	1 (2%)			
Brain cancer	3 (2%)		3 (5%)			
Ovary cancer	3 (2%)	1 (2%)	1 (2%)	1 (2%)		
Tongue base cancer	2 (1%)		1 (2%)	1 (2%)		
Rectal cancer	2 (1%)		1 (2%)	1 (2%)		
Anus/anal canal cancer	1 (1%)		1 (2%)			
Bronchus/lung cancer	1 (1%)		1 (2%)			
Cervix uteri cancer	1 (1%)		1 (2%)			
Bladder cancer	1 (1%)	1 (2%)				
Malignant neoplasm without specification of site	1 (1%)	1 (2%)				
Disease status (UICC) (*n* (%))					0.933	
I/II	102 (65%)	30 (67%)	36 (63%)	36 (64%)		
III/IV	56 (35%)	15 (33%)	21 (37%)	20 (36%)		
Chemotherapy						
Duration (weeks, mean ± SD)	17.2 ± 5.3	17.0 ± 5.2	16.7 ± 5.1	17.8 ± 5.7	0.672	
Time between last chemotherapy and post_0_ (days, mean ± SD)	22.9 ± 9.2	22.0 ± 9.3	23.5 ± 8.9	23.0 ± 9.4	0.411	
Taxane-based (*n* (%))	87 (53%)	27 (55%)	30 (53%)	30 (53%)	0.961^c^	
Taxane-platinum combination (*n* (%))	52 (32%)	15 (31%)	18 (32%)	19 (33%)		
Platinum-based (*n* (%))	19 (12%)	7 (14%)	6 (11%)	6 (11%)		
Vinca alkaloid (*n* (%))	4 (2%)		3 (5%)	1 (2%)		
Platinum-vinca alkaloid combination (*n* (%))	1 (1%)			1 (2%)		
Relative dose intensity						
Relative dose intensity (%, mean ± SD)	93.2 ± 8.6	94.5 ± 8.4	93.1 ± 8	92.2 ± 9.4	0.461	
min. 85% of planned dose intensity (*n* (%))	124 (85%)	41 (93%)	44 (81%)	39 (81%)	0.187	
Relative cumulative dose (%, mean ± SD)	93.9 ± 10.6	93.7 ± 12.7	93.9 ± 8.8	94.2 ± 10.4	0.395	
min. 85% of planned dose (*n* (%))	121 (81%)	38 (84%)	44 (81%)	39 (78%)	0.723	
Behavioural profile						
Smoking (*n* (%))					0.378	
Never smoker	66 (42%)	18 (37%)	27 (50%)	21 (38%)		
Former smoker	63 (40%)	19 (39%)	18 (33%)	26 (47%)		
Current smoker	29 (18%)	12 (24%)	9 (17%)	8 (15%)		
Alcohol consumption (WHO) (*n* (%))					0.697	
Non-drinker (0 g/day)	42 (26%)	12 (24%)	12 (22%)	18 (33%)		
Harmless use (f: ≤ 12 g/day, m: ≤ 24 g/day)	94 (59%)	29 (59%)	34 (62%)	31 (56%)		
Harmful use (f: > 12 g/day, m: > 24 g/day)	23 (14%)	8 (16%)	9 (16%)	6 (11%)		

*post*_*0*_ assessment point at the completion of neurotoxic chemotherapy.

Bold *P* values are considered statistically significant different (*P* < 0.05).

^a^No patients were included who already showed CIPN symptoms (see exclusion criteria).

^b^Breast cancer vs. others.

^c^Chemotherapy containing taxanes vs. others.

### Adherence to the interventions

Table S1a and S1b provide detailed information about exercise adherence. Briefly, the mean intervention length was 20 weeks for both groups. Mean attendance rate was 55% in the SMT and 49% in the RT group. The reasons for missed training sessions are listed in Table S3. Thirty-five patients were classified as adherent and included in PP/PP_EX_ analyses (SMT: *N* = 20, RT: *N* = 15). Non-adherent patients had lower physical and cognitive function as well as higher fatigue and insomnia baseline values on the EORTC QLQ-C30 subscales (all *P* < 0.024, Table S4).

Twenty-three patients reported mild training associated adverse events without indication for medical treatment (SMT: *N* = 10 (21%), RT: *N* = 13 (25%); see Table S5 for details).

During follow-up, 26% of the UC patients started a structured training: SMT (*N* = 6, mean attendance rate: 62.0%, range: 22.2–92.9%), RT (*N* = 7, mean attendance rate: 41.5%, range 23.1–64.3%), or endurance training (*N* = 1, attendance rate: 100%). Of these patients, only *n* = 4 (SMT *n* = 3, endurance training *n* = 1) were classified as “adherent” (at least 66.67% attendance rate). Reported exercise minutes per week increased descriptively in this group (post_0_-post_6_: +34.9 (−40 to 109.8), *P* = 0.359).

### CIPN signs and symptoms

Table 3 provides summarised data for CIPN signs/symptoms revealed by ITT analyses. Complementary values (e.g. analysed number of participants, ES for within-group comparisons), as well as results of secondary outcomes and complete PP/PP_EX_ analyses, are presented in Table S6.

**Table 3:** Intention-to-treat analysis: CIPN signs and symptoms.

	Group	pre	post_0_	post_3_	post_6_	pre–post_0_	Between-group comparison [pre–post_0_]			
Outcome		mean ± SD	mean ± SD	mean ± SD	mean ± SD	Adjusted^a^ mean change (95% CI)	Comparison	Adjusted^a^ between-group difference (95% CI)	*P* value	ES (95% CI)
*Total Neuropathy Score*										
TNSr [sum score, 0–36]	SMT	1.4 ± 2	7.2 ± 4.1	8.2 ± 4.2	7.1 ± 4	**4.5 (2.8 to 6.2)**	SMT vs. UC	0.3 (−1.6 to 2.3)	0.908	−0.15 (−0.57 to 0.27)
	RT	1.6 ± 1.5	7.6 ± 4.7	6.5 ± 4.3	6.8 ± 4.1	**4.7 (3.1 to 6.2)**	RT vs. UC	0.5 (−1.4 to 2.4)	0.809	−0.21 (−0.62 to 0.19)
	UC	2.3 ± 3.1	7.8 ± 4.7	7.6 ± 5.1	6.5 ± 4	**4.2 (2.5 to 5.8)**	SMT vs. RT	−0.2 (−2.2 to 1.8)	0.982	0.07 (−0.36 to 0.49)
TNSc [sum score, 0–28]	SMT	0.5 ± 1.2	4.9 ± 3.1	5.8 ± 3.5	5.3 ± 3.4	**3.2 (1.8 to 4.5)**	SMT vs. UC	−0.1 (−1.7 to 1.5)	0.983	0.08 (−0.33 to 0.48)
	RT	0.7 ± 1.1	5.6 ± 3.6	5.1 ± 3.6	5 ± 3.6	**3.7 (2.5 to 5.0)**	RT vs. UC	0.4 (−1.1 to 2)	0.773	−0.28 (−0.68 to 0.12)
	UC	1.4 ± 2.1	5.8 ± 3.7	5.7 ± 4.4	4.9 ± 4	**3.3 (2.0 to 4.6)**	SMT vs. RT	−0.6 (−2.1 to 1)	0.673	0.36 (−0.06 to 0.77)
TNSm [sum score, 0–24]	SMT	0.4 ± 1	4.2 ± 2.8	5.2 ± 3.2	4.8 ± 3.1	**2.8 (1.6 to 4.0)**	SMT vs. UC	−0.1 (−1.5 to 1.4)	0.995	0.04 (−0.37 to 0.45)
	RT	0.7 ± 1.1	5.1 ± 3.4	4.5 ± 3.2	4.4 ± 3.1	**3.6 (2.4 to 4.7)**	RT vs. UC	0.7 (−0.7 to 2.1)	0.438	−0.51 (−0.91 to −0.1)
	UC	1.2 ± 1.9	5 ± 3.2	4.9 ± 3.8	4.3 ± 3.5	**2.8 (1.6 to 4.0)**	SMT vs. RT	−0.8 (−2.2 to 0.6)	0.395	0.55 (0.13 to 0.97)
*Nerve conduction studies*										
CMAP (mV)	SMT	7.6 ± 3	5.7 ± 3	5.5 ± 2.5	6.4 ± 2.6	**−1.4 (−2.2 to −0.7)**	SMT vs. UC	0.1 (−0.9 to 1)	0.984	0.02 (−0.38 to 0.42)
	RT	8 ± 2.5	6.1 ± 2.7	5.7 ± 2.7	6.4 ± 2.6	**−1.3 (−1.9 to −0.6)**	RT vs. UC	0.2 (−0.6 to 1.1)	0.813	0.08 (−0.30 to 0.46)
	UC	7.4 ± 2.9	5.4 ± 2.4	6.2 ± 2.8	5.9 ± 2.7	**−1.5 (−2.2 to −0.8)**	SMT vs. RT	−0.2 (−1.1 to 0.8)	0.913	−0.06 (−0.45 to 0.34)
SNAP (µV)	SMT	10.8 ± 4.3	7.6 ± 3.9	7.2 ± 3.6	8 ± 3.8	**−3.2 (−4.5 to −1.8)**	SMT vs. UC	−0.6 (−2.3 to 1)	0.612	−0.14 (−0.55 to 0.26)
	RT	10.6 ± 4.3	8.1 ± 4.4	8.4 ± 4.1	8.2 ± 4.5	**−2.5 (−3.6 to −1.3)**	RT vs. UC	0.1 (−1.5 to 1.6)	0.996	0.01 (−0.38 to 0.40)
	UC	11.3 ± 5	8.2 ± 5.1	8.9 ± 5.1	9.2 ± 5.5	**−2.5 (−3.8 to −1.3)**	SMT vs. RT	−0.7 (−2.3 to 0.9)	0.562	−0.16 (−0.56 to 0.25)
NCV (peroneal) (m/s)	SMT	48 ± 3.7	46.8 ± 3.7	46.1 ± 4.3	46.3 ± 4.1	**−2.3 (−3.5 to −1.1)**	SMT vs. UC	−0.2 (−1.6 to 1.2)	0.943	−0.05 (−0.46 to 0.35)
	RT	48.8 ± 3.6	46.9 ± 4.1	48.2 ± 4.2	47.9 ± 3.8	**−2.2 (−3.2 to −1.2)**	RT vs. UC	−0.1 (−1.4 to 1.2)	0.988	−0.02 (−0.41 to 0.36)
	UC	48.7 ± 3.8	47.2 ± 4.4	47.3 ± 4.4	47.3 ± 3.8	**−2.1 (−3.2 to −1.0)**	SMT vs. RT	−0.1 (−1.5 to 1.3)	0.981	−0.03 (−0.44 to 0.38)
NCV (sural) (m/s)	SMT	48.4 ± 5	46.4 ± 4.8	46.8 ± 4.7	45.8 ± 5.2	**−3.1 (−5.2 to −0.9)**	SMT vs. UC	0 (−2.7 to 2.6)	0.999	−0.01 (−0.42 to 0.40)
	RT	48 ± 4.7	45.9 ± 5.9	45.8 ± 6.1	45.6 ± 4.8	**−3.1 (−5.0 to −1.2)**	RT vs. UC	−0.1 (−2.6 to 2.4)	0.997	−0.01 (−0.41 to 0.38)
	UC	48.4 ± 6.5	46.3 ± 5.3	45.3 ± 3.6	46.1 ± 3.9	**−3.0 (−5.1 to −1.0)**	SMT vs. RT	0.0 (−2.6 to 2.7)	1.00	0.00 (−0.41 to 0.42)
*Patient-reported CIPN symptoms [EORTC QLQ-CIPN20]*										
CIPN-15 [sum score, 0–100]	SMT	1.9 ± 4	14.2 ± 14.5	15.2 ± 19.1	12.1 ± 13.5	**9.8 (3.7 to 16.0)**	SMT vs. UC	2.2 (−5.2 to 9.5)	0.761	−0.47 (−0.87 to −0.07)
	RT	2.2 ± 3.5	15.4 ± 17.9	14.4 ± 16.3	12.1 ± 14.3	**10.4 (5.1 to 15.7)**	RT vs. UC	2.7 (−4.3 to 9.7)	0.624	−0.59 (−0.98 to −0.2)
	UC	3.6 ± 5.9	14.3 ± 15.3	14.8 ± 18.7	13.3 ± 17.2	**7.7 (2.0 to 13.4)**	SMT vs. RT	−0.6 (−7.9 to 6.8)	0.983	0.12 (−0.28 to 0.52)
Sensory symptoms feet [sum score, 0–100]	SMT	2 ± 4.9	17.5 ± 18	18.8 ± 22.4	14.4 ± 18.2	**9.5 (1.6 to 17.3)**	SMT vs. UC	−1.5 (−10.9 to 7.9)	0.925	0.23 (−0.17 to 0.63)
	RT	1.4 ± 3.3	19.4 ± 21.1	18.8 ± 22.2	17.1 ± 20.2	**11.9 (5.0 to 18.7)**	RT vs. UC	0.9 (−8.2 to 10)	0.971	−0.14 (−0.52 to 0.24)
	UC	4.2 ± 9.3	21.5 ± 21.7	21 ± 23.4	18.4 ± 23.1	**11 (3.6 to 18.3)**	SMT vs. RT	−2.4 (−11.8 to 7)	0.819	0.37 (−0.03 to 0.77)
Motor symptoms feet [sum score, 0–100]	SMT	2.9 ± 6.3	10.4 ± 13.7	14.7 ± 21.6	8.5 ± 12.7	4.8 (−1.0 to 10.7)	SMT vs. UC	1.4 (−5.6 to 8.4)	0.885	−0.17 (−0.56 to 0.23)
	RT	2.8 ± 4.9	12.3 ± 15.8	12.9 ± 17.7	11.5 ± 15.4	**6.6 (1.6 to 11.7)**	RT vs. UC	3.2 (−3.5 to 9.9)	0.499	−0.38 (−0.76 to 0.01)
	UC	6.1 ± 11.9	12.3 ± 18.2	14.2 ± 24	13.4 ± 23	3.5 (−2.0 to 8.9)	SMT vs. RT	−1.8 (−8.8 to 5.2)	0.815	0.21 (−0.18 to 0.61)
Symptoms hands [sum score, 0–100]	SMT	2 ± 5.1	15.4 ± 16.2	15 ± 19.8	13 ± 14.4	**12.5 (5.7 to 19.2)**	SMT vs. UC	4.6 (−3.5 to 12.6)	0.373	−0.69 (−1.1 to −0.28)
	RT	3.2 ± 6.3	15.6 ± 19.9	14.2 ± 16.4	11.1 ± 16	**11.1 (5.3 to 17.0)**	RT vs. UC	3.2 (−4.4 to 10.9)	0.582	−0.49 (−0.87 to −0.1)
	UC	3.5 ± 8	12.8 ± 15.3	13.1 ± 19.6	11.7 ± 14.6	**7.9 (1.6 to 14.2)**	SMT vs. RT	1.3 (−6.7 to 9.4)	0.918	−0.20 (−0.6 to 0.19)

*CIPN-15* sum score based on 15 items of the EORTC QLQ-CIPN20 questionnaire, *CMAP* compound muscle action potential of the peroneal nerve, *NCV* nerve conduction velocity, *pre* assessment point before neurotoxic chemotherapy, *post*_*0*_ assessment point 3 weeks after neurotoxic chemotherapy, *post*_*3*_ assessment point 3 months after post_0_, *post*_*6*_ assessment point 6 months after post_0_, *SD* standard deviation, *SNAP* sensory nerve action potential of sural nerve, *TNSc* Total Neuropathy Score (clinical), *TNSm* Total Neuropathy Score (modified), *TNSr* Total Neuropathy Score (reduced).

The table shows descriptive statistics of clinically, electrophysiologically assessed and subjectively perceived CIPN signs and symptoms for all assessment points. Adjusted mean change (within groups) and between-group differences are only presented for intervention time as revealed by intention-to-treat analyses. Bold value indicates statistical significance at the level of 5%.

^a^Regression models were adjusted for baseline value, sex, age, and therapy-randomisation strata.

**Fig. 3:**
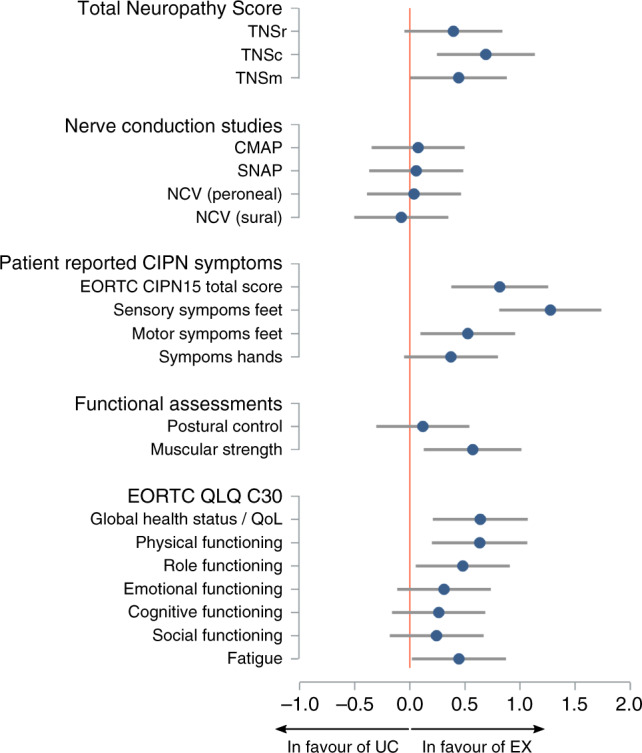
Effect sizes for CIPN signs and symptoms and other symptoms associated with anticancer therapy (pre–post_0_, PP_EX_ analysis). *CMAP* compound muscle action potential of peroneal nerve, *NCV* nerve conduction velocity, *QoL* quality of life, *SNAP* sensory nerve action potential of sural nerve, *TNSc* Total Neuropathy Score (clinical), *TNSm* Total Neuropathy Score (modified), *TNSr* Total Neuropathy Score (reduced).

Overall, the TNSr score increased significantly in all three groups during chemotherapy with small, non-significant between-group differences; comparable results were found for the TNS variations (TNSc, TNSm) and NCS parameters (ITT, Table 3). PP/PP_EX_ analyses provided comparable results with larger effect sizes (Fig. 3).

During the intervention period, between-group comparisons of the increased EORTC CIPN-15 total and sub-scores revealed no significant differences between groups (ITT). PP_EX_ analysis showed a significant between-group comparison in favour of EX regarding sensory symptoms in the feet (PP_EX_ pre–post_0_: *P* = 0.039, ES = −1.27).

During follow-up, TNSr and its variations, as well as NCS parameters, did not change according to inferential statistics. EORTC CIPN-15 total score revealed a significant decrease in SMT and RT (ITT post_0_–post_6_: *P* < 0.038) and for symptoms in the hands for all groups (ITT post_0_–post_6_: *P* < 0.045). Between-group comparisons revealed marginal, non-significant differences with overall small effect sizes during the follow-up periods. PP and PP_EX_ analyses showed overall comparable results.

### Functional assessments

COP mean velocity in BP_EC_ increased significantly during the intervention period in all groups (ITT pre–post_0_: *P* *<* 0.015). PP showed a tendency towards unchanged adjusted mean change values for SMT (pre–post_0_: *P* = 0.235). For all analyses approaches, between-group differences were non-significant with small effect sizes. During follow-up, all groups showed decreased COP mean velocities (ITT post_0_–post_6_: *P* < 0.019), however, this effect only remained significant for the SMT group in both PP analyses. Between-group differences were non-significant and had small effect sizes for all analyses.

In all analyses approaches, mean MP_EO_ standing time remained unchanged for SMT and RT, but significantly decreased in UC, resulting in a significant between-group comparison for ITT analysis in favour of both exercise groups (pre–post_0_: SMT vs. UC *P* = 0.045, ES = 0.27; RT vs. UC *P* = 0.023, ES = 0.28). During follow-up, comparisons did not reveal any significant differences (ITT, PP, PP_EX_).

RT and SMT sustained their baseline muscle strength status while UC showed decreased values (ITT pre–post_0_: *P* = 0.016). PP analyses revealed a significant gain of muscle strength for RT (pre–post_0_: *P* = 0.003) and PP_EX_ analysis for adherent EX (pre–post_0_: *P* = 0.027). Consequently, between-group comparisons revealed a significant difference in favour of RT compared to UC (pre–post_0_: ITT *P* = 0.045, ES = 0.30; PP *P* < 0.001, ES = 0.81) as well as for SMT compared to UC (PP pre–post_0_: *P* = 0.041, ES = 0.38) and for EX compared to UC (PP_EX_ pre–post_0_: *P* < 0.001, ES = 0.57). During follow-up, no significant between-group comparisons and overall small effect sizes were found.

### Patient-reported outcomes

During the intervention period, primary ITT analyses of the EORTC QLQ-C30 scores revealed non-significant between-group differences and mainly small effect sizes. However, PP analyses showed significant group differences between RT and UC for global health status (pre–post_0_: *P* = 0.018, ES = 0.85) and social functioning (pre–post_0_: *P* = 0.047, ES = 0.52). PP_EX_ analyses additionally showed significant between-group differences in favour of adherent EX for physical functioning (pre–post_0_: *P* = 0.014, ES = 0.63), role functioning (pre–post_0_: *P* = 0.02, ES = 0.48) and fatigue (pre–post_0_: *P* = 0.016, ES = 0.45), and borderline significance for pain (pre–post_0_: *P* = 0.057, ES = 0.32). Overall, the follow-up period revealed mainly non-significant between-group comparisons with small effect sizes for all analyses approaches.

Fear of falling increased in UC during chemotherapy (ITT pre–post_0_: *P* = 0.037, ES = 0.57), but not in SMT and RT. However, pre–post_0_ changes did not differ between groups in all analyses approaches, nor did the number of falls during the intervention period (see Table S7 for details).

### Chemotherapy completion rate

In the ITT study sample, chemotherapy dose reductions and early terminations were evenly distributed between groups and most often associated with CIPN symptoms (Table S8). Mean RDI did not differ between study groups (ITT *P* = 0.461, Table 2; PP: SMT 97.3 ± 3.8, RT 95.7 ± 6.0, UC 92.2 ± 9.4; *P* = 0.103), except when comparing EX with UC (PP_EX_: EX: 96.6 ± 4.8, UC: 92.2 ± 9.4; *P* = 0.045). So did the clinically relevant threshold of 85% RDI (EX: 94%, UC: 76%; *P* = 0.032). Concomitant CIPN prevention or treatment measures did not differ between groups (Table S8).

## Discussion

The PIC study aimed to investigate the preventive effect of sensorimotor exercise training (SMT) or resistance training (RT) versus usual care (UC) on CIPN during neurotoxic chemotherapy. Our primary ITT analysis revealed that none of the exercise programmes was able to impact the progression of neurologically objectified and patient-reported CIPN signs/symptoms. Due to the high numbers of missed training sessions in both groups, we excluded non-adherent patients for exploratory per-protocol analyses. Subjectively perceived sensory symptoms in the feet increased less during chemotherapy in the adherent exercisers (pooled group: SMT+RT) compared to UC. Furthermore, compliance to chemotherapy was found to be enhanced in this group. On the functional level, we identified a better course of muscular strength in favour of the adherent exercisers, as well as better results in terms of overall quality of life, physical and role functioning, fatigue, and a trend-level effect for pain.

Only a few RCTs have investigated the preventive effect of exercise on CIPN during neurotoxic chemotherapy [16–21], of which only two used clinical instruments to assess CIPN symptoms [16, 19]. In accordance with our ITT results, Bland et al. [19] did not demonstrate an intervention effect of a multimodal exercise programme during taxane-based chemotherapy with regard to quantitative sensory tests (deep sensitivity: tuning fork; pain: pinprick). In contrast, a sub-analysis of a comparable exercise programme showed a reduction of CIPN symptoms by tuning fork evaluation in the intervention group but not in the control group [16].

Similarly, the results of the other studies regarding subjectively perceived CIPN symptoms are largely consistent with our ITT analyses. The studies which used psychometrically tested questionnaires, that focus on CIPN symptoms in the whole body, were not able to find a significant intervention effect (EORTC QLQ-CIPN15 [19]) or only observed a trend-level effect (FACT/GOG-Ntx [20]). Kleckner et al. [17] used a numeric-rating-scale (NRS 0-10), which only focused on two symptom combinations in hands and feet. The authors reported a trend-level effect for the perception of numbness/tingling and a significant intervention effect for hot/coldness in favour of the intervention group. Comparable results were observed for adherent exercisers within our PP_EX_ analysis, who developed less sensory symptoms in the feet during chemotherapy compared to UC. A sub-analysis by Bland et al. [19] mirrors these findings by showing that multimodal exercise can prevent the progression of moderate-to-severe numbness in toes and feet within the first three taxane cycles. Based on the studies cited, it could be hypothesised that a preventive effect for CIPN signs and symptoms can only be detected in the body regions that are targeted by the training implemented, e.g. due to neural adaptations [37], release of neurotrophic factors [38] or reduced inflammation processes [39]. In our opinion, this is a highly relevant finding, since CIPN-induced dose modifications of chemotherapy are mainly based on patients’ subjective perception. Therefore, the better chemotherapy tolerance (mean RDI) observed in the adherent exercisers (97%) compared to UC (92%) may be associated with the shown lower perceived CIPN symptoms in this group. Although the evidence does not yet allow final conclusions to be drawn as to whether exercise actually has a positive influence on chemotherapy tolerance [40], these findings are in line with Bland et al. [19] and point towards a promising direction.

### Functional status and patient-reported outcomes

Various studies have shown that neurotoxic chemotherapy can have a negative effect on postural control [28, 41, 42], which may be partly prevented by a multimodal training programme [18]. Our COP data did not replicate this result and showed only a marginal trend in favour of the SMT group. Based on the mean standing time in MP_EO_ position, however, SMT and RT showed a more favourable progression of postural control than UC. The improved standing time in the RT group during neurotoxic chemotherapy could have been achieved by increased muscle strength observed in the RT-adherent patients [43]. Since cancer patients normally show a chemotherapy-induced deterioration of muscle strength [44]—as also shown in our UC group—the increase but also the maintenance of muscle strength by RT or SMT is an important finding.

Although CIPN and associated poor postural stability are known to increase the risk of falling [5, 7], the fall prevalence of 8% in our total cohort during chemotherapy is markedly lower compared with another study showing annual fall rates of 43–57% after cancer treatment [45]. This difference might be explained by the higher age of these patients (+10 years), but also by the longer time after diagnosis (+6 years). In addition, an enhanced focus on locomotion in everyday life due to the acute change in sensory perception and generally less everyday activities during chemotherapy may further explain this difference. The latter point might also be in line with the majority of our patients (71%) reporting low concerns about falling during chemotherapy (FES-I value <20) [46].

Finally, the adherent exercisers were able to enhance QoL during chemotherapy. The difference compared with UC (+12.9 points) can be seen as clinically meaningful [47], and is in accordance with Bland et al. [19]. In addition, we observed better results in favour of adherent exercisers in terms of physical and social functioning as well as fatigue and a trend-level effect for pain which are in line with a large body of exercise oncology studies [34].

### CIPN signs and symptoms during follow-up

Neurologically objectified CIPN signs/symptoms did not change during the follow-up period of 6 months, whereas EORTC CIPN-15 total score decreased significantly in RT and SMT as well as CIPN symptoms in the hands in all groups. However, group means were still elevated compared to baseline values. These results are in line with many other studies addressing the long-term persistence of CIPN symptoms after completion of chemotherapy [48]. Structured exercise interventions helped to positively influence objectively assessed [10–12] and subjectively perceived CIPN signs/symptoms [10, 12–14]. However, the proportion of patients who followed a structured exercise programme within our study and their adherence were probably too small to show this effect.

### Limitations and future directions

In comparison to most exercise intervention studies focusing on CIPN prevention, we provide the largest sample size for ITT and PP analyses with comprehensive and recommended CIPN diagnostics [49]. However, in line with Bland et al. [19], we were unable to achieve our target sample size and thus could not confirm our initial hypothesis through our primary ITT analyses. In addition to the lack of power, the high non-attendance rate in both exercise interventions, accompanied by a resulting insufficient training stimulus, could also explain the absence of an intervention effect. Therefore, we excluded non-adherent patients from analyses. Although most of the PP results show high effect sizes and are in line with other studies, these analyses are per se not confirmatory. Hence, the presented results need to be verified by future studies, not least to rule out a potential selection bias of the PP population, i.e. by showing that the higher training adherence and no other factors led to the intervention effects shown. Overall, future studies should amend the following aspects: (i) larger sample size of adherent exercisers, e.g. by means of measures to increase exercise adherence (see “Practical considerations”), (ii) focus on expanding recruitment to other entities, as most of our patients (and those in the other studies) had breast cancer and were female, which hampers generalisability, (iii) longer follow-up period with a larger sample size and comprehensive (device-based) physical activity monitoring, (iv) higher CIPN assessment density during chemotherapy in order to detect variations in the effectiveness of exercises [19] and consequently to be able to make adjustments and (v) modification of CIPN diagnostics towards several specifically tailored procedures that focus on the targeted training region instead of depicting the entire peripheral nerve status as our primary endpoint, TNSr. The latter point also implies the need for the validation of these modified CIPN assessments, as the results shown by us and Bland et al. [19] are based on a standardised questionnaire but not on psychometrically tested subscales.

### Practical considerations

Based on our results and those of other authors [16, 17, 19], it might be advisable to recommend a multimodal training approach to preventively influence as many facets of CIPN as possible. This multimodal training approach should consist of SMT and RT [16, 19], and possibly also endurance training [16, 17, 19]. However, this training approach can only be effective if an adequate training stimulus is achieved through sufficient exercise adherence. Almost half of the missed training sessions were due to side effects of anticancer treatment (e.g. nausea, vomiting, pain). It is conceivable that a temporary adjustment of the training (e.g. reduction of the duration and/or intensity) depending on the side effects that occurred may improve the training adherence. For example, Bland et al. [19] reduced the intensity of the supervised training in the first week after chemotherapy and were able to achieve a higher attendance rate (78 ± 23%). Nevertheless, some side effects will always require a (temporarily) abstinence from exercise (e.g. thrombosis). Therefore, it is even more important that patients miss as few training sessions as possible due to other reasons.

Approximately one-third of all missed training sessions in our study were based on time constraints and motivational issues. Adherence enhancing measures, which go beyond the conducted telephone calls and include well-founded behavioural change techniques, may additionally increase exercise adherence and thus the prevention effect in terms of perceived CIPN symptoms and functional limitations [50]. These might also help the non-adherent patients who had lower physical and cognitive function as well as higher fatigue and insomnia values at baseline compared to the adherent patients, to enhance their attendance rate.

## Conclusion

SMT and/or RT might be effective strategies to prevent sensory CIPN symptoms in the feet during neurotoxic chemotherapy and enhance chemotherapy tolerance as well as QoL. However, as these results are based on PP analysis, future studies need to confirm these findings.

## Supplementary information

Figure S1. Selected sensorimotor exercise training cards.

Table S1a. Sensorimotor exercise training: Prescribed and actual exercise dose and adherence outcomes.

Table S1b. Resistance training: Prescribed and actual exercise dose and adherence outcomes.

Table S2. Detailed information of cancer stages.

Table S3. Reasons for missed training sessions.

Table S4. Patient characteristics and significant baseline differences between adherent and non-adherent exercisers.

Table S5. Adverse events related to the exercise programs.

Table S6. Results revealed by ANCOVA for ITT, PP and PP_EX_ analyses. [Excel Table with three different sheets]

Table S7. Number of falls.

Table S8. Information on chemotherapy dose modifications, early termination and concomitant CIPN treatment and prevention measures.

## Data Availability

All raw data analysed for this publication can be made available on request.
