# Supplementary material for: Preventive effect of sensorimotor exercise and resistance training on chemotherapy-induced peripheral neuropathy: a randomised-controlled trial
Source: Br J Cancer. 2021 Jul 5;125(7):955–65. doi: 10.1038/s41416-021-01471-1 (PMC8476560; doi:10.1038/s41416-021-01471-1)
Supplement: Supplementary file 1 — Figure S1. Selected sensorimotor exercise training cards. [file 41416_2021_1471_MOESM1_ESM.pdf]

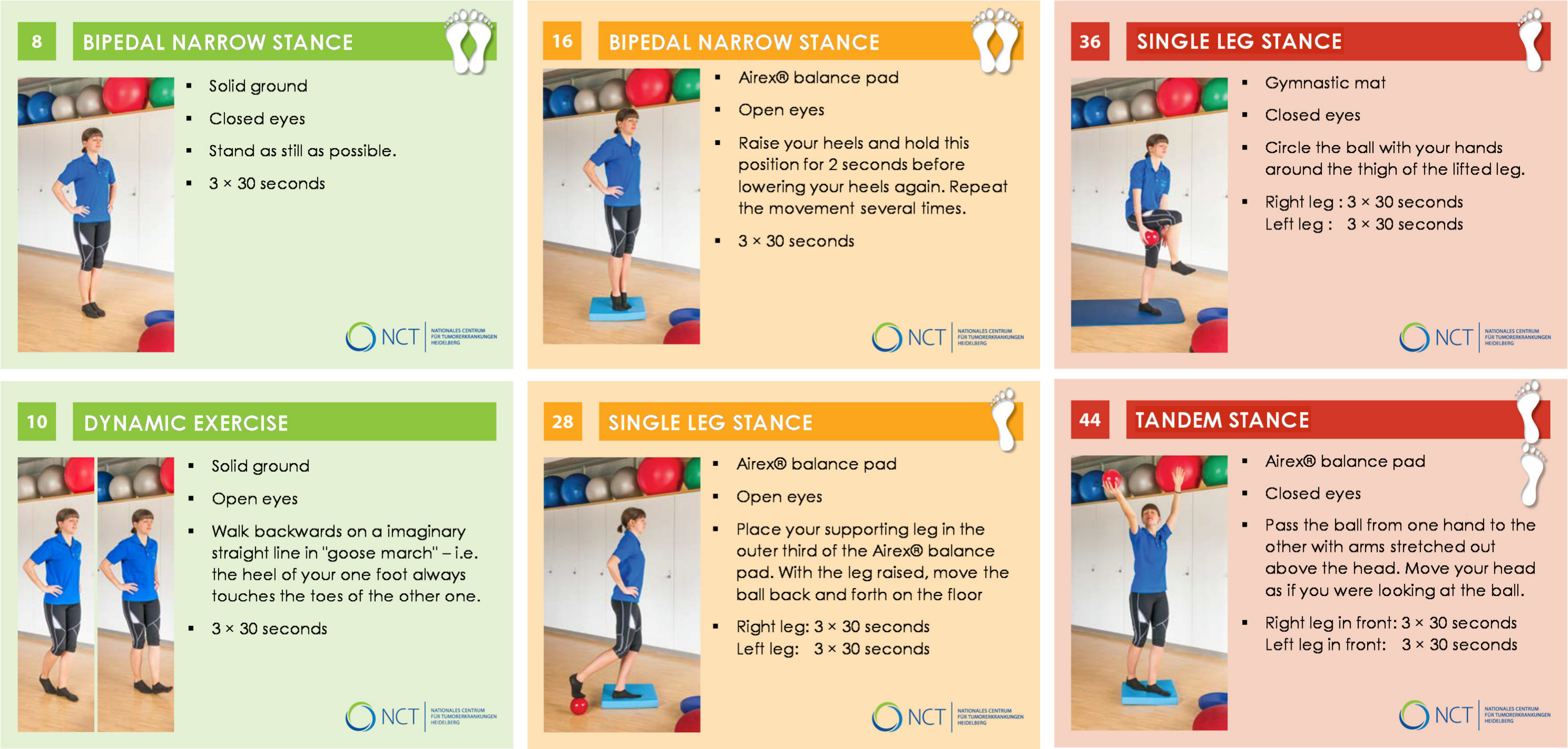

Figure S1. Selected sensorimotor exercise training cards. The exercises primarily included static balance exercises in an upright position – every fifth exercise was of dynamic character. For progression purposes, the exercises varied in the following aspects: base of support (e.g. bipedal vs. monopedal stance), surface (e.g. solid ground vs. Airex Balance Pad), head position (e.g. straight vs. head back), and visual control (open vs. closed eyes) and were combined with additional tasks if possible (e.g. throwing a ball). The patients were instructed to increase exercises difficulty in each training session and over the entire training period from easy to difficult (the higher the number in the left corner of a card, the more difficult the exercise). Every exercise was rated on a numeric rating scale after it was carried out (NRS: 1 - very easy, 2 - easy, 3 - intermediate, 4 - difficult, 5 - very difficult). If an exercise was too easy (NRS 1 or 2), the training card was removed from the catalogue in order to continue with more difficult exercises in the following training sessions.
