# Supplementary material for: Preventive effect of sensorimotor exercise and resistance training on chemotherapy-induced peripheral neuropathy: a randomised-controlled trial
Source: Br J Cancer. 2021 Jul 5;125(7):955–65. doi: 10.1038/s41416-021-01471-1 (PMC8476560; doi:10.1038/s41416-021-01471-1)
Supplement: Supplementary file 2 — Table S1a. Sensorimotor exercise training: Prescribed and actual exercise dose and adherence outcomes. [file 41416_2021_1471_MOESM2_ESM.pdf]

Table S1a. Sensorimotor exercise training: Prescribed and actual exercise dose and adherence outcomes.

	PRESCRIBED	ACTUAL	
FREQUENCY	3x/week	▪ Attended training weeks [% of planned length]	68.1 ± 29.3
		▪ Attended training sessions [% of planned sessions] [o/w supervised]	53.3 ± 27.5 [4.6 ± 12.2]
		▪ Treatment interruption [n per patient] [1]	1.5 ± 1.6
		▪ Length of treatment interruption [weeks]	2.5 ± 1.4
		▪ Permanent treatment discontinuation [n] [2]	11 (23%)
INTENSITY	various difficulties<br>progression: based on individual perception (see Figure EX1 for details)	▪ Missed progress [% of attended training sessions] [3]	19.1 ± 14.7
		▪ Sessions requiring dose reduction(s) [% of attended training sessions] [4]	23.9 ± 15
TIME	total length according to CHT regime plus 3 weeks [until $post_0$]<br>3x35 min/week	▪ Total length [weeks]	20.7 ± 4.7
		▪ Total training duration per week [min]	81.5 ± 21.7
		▪ Number of exercises per session [mean]	7.8 ± 1.9
TYPE	**Sensorimotor exercise training**<br>STATIC AND DYNAMIC (16%) EXERCISES IN UPRIGHT POSITION FOR IMPROVING POSTURAL CONTROL/BALANCE. EXERCISES WERE PROGRESSIVELY DESIGNED AND VARIED REGARDING BASE OF SUPPORT, SURFACE, HEAD POSITION, VISUAL CONTROL, AND ADDITIONAL TASKS. EACH EXERCISE WAS PERFORMED 3×30 SECONDS WITH AT LEAST 30 SECONDS PAUSE BETWEEN SETS.		

The adherence outcomes are presented as mean ± SD (unless otherwise indicated) and are based on the data of 48 patients. Four patients out of 52 (8%) did not start their assigned sensorimotor exercise training due to: study exclusion (n=3, see flow-chart), an unplanned inpatient admission at the beginning of the study made the patient feel that the additional training program was too much (n=1). Abbreviations and additional explanations/definitions: [1] missing at least three consecutive sessions (Nilsen et al. 2018 Med Sci Sports Exerc); [2] permanent discontinuation of exercise intervention within the first two thirds of the planned duration; [3] patients indicated that at least one of the performed exercises was very easy or easy (NRS 1 or 2) without increasing the difficulty in the following training sessions (i.e. using at least one training card with a higher number); [4] patients indicated that the average dose/difficulty of a training session was reduced, i.e. the mean value of the card numbers has decreased from one training session to the next.
