# Supplementary material for: Preventive effect of sensorimotor exercise and resistance training on chemotherapy-induced peripheral neuropathy: a randomised-controlled trial
Source: Br J Cancer. 2021 Jul 5;125(7):955–65. doi: 10.1038/s41416-021-01471-1 (PMC8476560; doi:10.1038/s41416-021-01471-1)
Supplement: Supplementary file 3 — Table S1b. Resistance training: Prescribed and actual exercise dose and adherence outcomes. [file 41416_2021_1471_MOESM3_ESM.pdf]

**Table S1b.** Resistance training: Prescribed and actual exercise dose and adherence outcomes.

| | PRESCRIBED | | ACTUAL | | |
|---|---|---|---|---|---|
| | SUPERVISED | HOME-BASED | | SUPERVISED | HOME-BASED |
| FREQUENCY | 2x/week | 1x/week | ▪ Attended training weeks [% of planned length] | 55.1 ± 28.4 | 55.1 ± 29.9 |
| | | | ▪ Attended training sessions [% of planned sessions] | 42.7 ± 25.7 | 55.1 ± 29.9 |
| | | | ▪ Treatment interruption [n per patient] [1] | 2.0 ± 1.9 | |
| | | | ▪ Length of treatment interruption [weeks] | 2.3 ± 1.0 | |
| | | | ▪ Permanent treatment discontinuation [n] [2] | 10 (19%) | |
| INTENSITY | start at 70-80%1RM progression: 3×12 repetitions in three consecutive training sessions | RPE 14-16 | ▪ Mean intensity over time | 68.4 ± 15.4 [3] | 14.4 ± 1.7 [4] |
| | | | ▪ Training weight increases [%] [5] | 19.9 ± 13.4 | *n/a* |
| | | | ▪ Training weight reductions [%] [5] | 9.7 ± 10.9 | *n/a* |
| | | | ▪ Missed progress [6] | 70.3 ± 26.9 | *n/a* |
| TIME | total length according to CHT regime plus 3 weeks [until $post_0$] | | ▪ Total length [weeks] | 20.3 ± 5.5 | |
| | 2x 45 min/week [8 exercises/session] | 15 min/week [3 exercises/session] | ▪ Total training duration per week [min] | 68.6 ± 17.8 | 17 ± 6.1 |
| | | | ▪ Numbers of exercises per session [median] | 8.3 ± 1.4 | 2.8 ± 0.4 |
| TYPE | **Supervised machine-based resistance training + home-based core stability exercises**<br>SUPERVISED: LEG PRESS, KNEE EXTENSION AND FLEXION, ROWING, LATERAL PULL DOWN, SHOULDER INTERNAL AND EXTERNAL ROTATION, BUTTERFLY, BUTTERFLY REVERSE<br>HOME-BASED: 15 DIFFERENT CORE STABILITY EXERCISES IN VARIOUS POSITIONS: SUPINE POSITION, PRONE POSITION, QUADRUPED POSITION, PLANK POSITION | | | | |

The adherence outcomes are presented as mean ± SD (unless otherwise indicated) and are based on the data of 54 patients. Seven patients out of 60 (12%) did not start their assigned resistance training program due to: time constraints (n=3), study exclusion (see flow-chart, n=2), medical contraindication (port thrombosis, n=1), training contents did not please (n=1). Abbreviations and additional explanations/definitions: [1] missing at least three consecutive sessions (Nilsen et al. 2018 Med Sci Sports Exerc); [2] permanent discontinuation of exercise intervention within the first two thirds of the planned duration; [3] %1RM [percent of one repetition maximum]; [4] BORG Rating of Perceived Exertion (RPE); [5] number of training weight increases/reductions per exercise (compared to the previous training session) in relation to the total number of exercises during the intervention period; [6] training weight was not increased after patient moved the target weight in 3×12 repetitions in three consecutive training sessions.
