# Supplementary material for: Preventive effect of sensorimotor exercise and resistance training on chemotherapy-induced peripheral neuropathy: a randomised-controlled trial
Source: Br J Cancer. 2021 Jul 5;125(7):955–65. doi: 10.1038/s41416-021-01471-1 (PMC8476560; doi:10.1038/s41416-021-01471-1)
Supplement: Supplementary file 4 — Table S2. Detailed information of cancer stages. [file 41416_2021_1471_MOESM4_ESM.pdf]

Table S2. Detailed information of cancer stages according to UICC [n (%)].

	Total	SMT	RT	UC
1	8 (5%)	4 (8%)	2 (4%)	2 (4%)
1A	32 (20%)	9 (18%)	9 (16%)	14 (25%)
1B	1 (1%)	1 (2%)		
1C	1 (1%)		1 (2%)	
2	2 (1%)		1 (2%)	1 (2%)
2A	33 (20%)	7 (14%)	14 (25%)	12 (21%)
2B	23 (14%)	9 (18%)	7 (12%)	7 (12%)
3	7 (4%)	3 (6%)	3 (5%)	1 (2%)
3A	15 (9%)	4 (8%)	5 (9%)	6 (11%)
3B	6 (4%)	1 (2%)	3 (5%)	2 (4%)
3C	5 (3%)	1 (2%)	3 (5%)	1 (2%)
4	14 (9%)	4 (8%)	4 (7%)	6 (11%)
4A	5 (3%)		2 (4%)	3 (5%)
4B	3 (2%)	2 (4%)		1 (2%)
WHO II°	2 (1%)		2 (4%)	
WHO III°	1 (1%)		1 (2%)	
unknown	5 (3%)	4 (8%)		1 (2%)
