# Supplementary material for: Preventive effect of sensorimotor exercise and resistance training on chemotherapy-induced peripheral neuropathy: a randomised-controlled trial
Source: Br J Cancer. 2021 Jul 5;125(7):955–65. doi: 10.1038/s41416-021-01471-1 (PMC8476560; doi:10.1038/s41416-021-01471-1)
Supplement: Supplementary file 5 — Table S3. Reasons for missed training sessions. [file 41416_2021_1471_MOESM5_ESM.pdf]

Table S3. Reasons for missed training sessions.

	SMT	RT
**Somatic side effects of anticancer treatment [%]**	**27.6**	**34.4**
- nausea / vomiting / diarrhoea	11.3	14.5
- pain	9.9	7.8
- thrombosis	4.6	2.3
- skin reactions	0.9	2.5
- hematopoietic disturbances	0.2	1.7
- cardiac signs	0.0	0.3
- visual impairments	0.0	1.2
- CIPN	0.0	0.5
- not further specified	0.7	3.6
**Biopsychosocial side effects of anticancer treatment [%]**	**20.2**	**14.4**
- fatigue	14.9	7.5
- feeling unwell	5.1	6.4
- mental constrains	0.2	0.5
**Other medical issues related to anticancer treatment [%]**	**2.1**	**4.1**
- complications with subcutaneous port	1.4	2.7
- hospitalization / post-operation	0.7	1.4
**Others**	**50.1**	**47.1**
- time constraints	25.8	23.2
- motivation	11.3	5.3
- infection	7.4	3.6
- organisational reasons	5.1	12.5
- orthopaedic contraindications	0.5	1.9
- not further specified	0.0	0.6
