# Supplementary material for: Preventive effect of sensorimotor exercise and resistance training on chemotherapy-induced peripheral neuropathy: a randomised-controlled trial
Source: Br J Cancer. 2021 Jul 5;125(7):955–65. doi: 10.1038/s41416-021-01471-1 (PMC8476560; doi:10.1038/s41416-021-01471-1)
Supplement: Supplementary file 6 — Table S4. Patient characteristics and significant baseline differences between adherent and non-adherent exercisers. [file 41416_2021_1471_MOESM6_ESM.pdf]

Table S4. Patient characteristics and significant baseline differences between adherent and non-adherent exercisers.

	Adh. EX	Non-adh. EX	p-value
**Patient characteristics**			
N	35	71	-
Sex [f:m, n]	27:8	62:9	.179
Age [mean ± SD]	52.1 ± 10.9	52.9 ± 11.5	.752
BMI [mean ± SD]	26 ± 5.1	26.7 ± 5.2	.573
Married [n (%)]	29 (74%)	52 (85%)	.245
Completed university [n (%)]	16 (30%)	21 (47%)	.088
Breast cancer [n (%)]	25 (71%)	52 (73%)	.844
Stage III/IV [n (%)]	12 (36%)	24 (35%)	.875
**EORTC QLQ C30 [baseline values, mean ± SD]**			
Physical functioning	91.8 ± 15.4	88.1 ± 13.6	.024 *
Cognitive functioning	88.2 ± 21.1	78.6 ± 20.9	.007 *
Fatigue	21.4 ± 23.7	36.0 ± 26.2	.004 *
Insomnia	24.5 ± 28.8	43.8 ± 34.3	.006 *

We tested all primary and secondary outcome variables, but only the significant differences are shown besides patient characteristics. Abbreviations: ahd., adherent; EX, exercisers.
