# Supplementary material for: Preventive effect of sensorimotor exercise and resistance training on chemotherapy-induced peripheral neuropathy: a randomised-controlled trial
Source: Br J Cancer. 2021 Jul 5;125(7):955–65. doi: 10.1038/s41416-021-01471-1 (PMC8476560; doi:10.1038/s41416-021-01471-1)
Supplement: Supplementary file 7 — Table S5. Adverse events related to the exercise programs. [file 41416_2021_1471_MOESM7_ESM.pdf]

Table S5. Adverse events related to the exercise programs.

		SMT	RT
Patients reporting at least one AE [n (%)]		10 (21%[1])	13 (25%[1])
**Adverse events**			
Pain	▪ after 1RM test	-	2
	▪ musculoskeletal	4	14
	▪ other	1	3
Fatigue		2	4
Dizziness		9	2

Note: [1] The percentage is based on the number of patients who ever exercised during the intervention period (SMT n=48; RT n=53).
