# Supplementary material for: Preventive effect of sensorimotor exercise and resistance training on chemotherapy-induced peripheral neuropathy: a randomised-controlled trial
Source: Br J Cancer. 2021 Jul 5;125(7):955–65. doi: 10.1038/s41416-021-01471-1 (PMC8476560; doi:10.1038/s41416-021-01471-1)
Supplement: Supplementary file 9 — Table S7. Number of falls. [file 41416_2021_1471_MOESM9_ESM.pdf]

Table S7. Number of falls.

	**pre**	**pre-post$_0$**	**post$_0$-post$_3$$^+$**	**post$_3$-post$_6$$^+$**
SMT [n (%)]	2 (4%)	5 (11%)	4 (10%)	5 (12%)
RT [n (%)]	6 (11%)	2 (4%)	1 (2%)	1 (2%)
UC [n (%)]	4 (7%)	6 (11%)	3 (6%)	4 (9%)
p-value	.485	.290	.280	.047

Table S7 shows the number of falls according to different time periods within the PIC study. The first time period (pre) refers to the 12 months prior to study inclusion. **Note**: The follow up time-periods marked with a "+" have high numbers of missing values (post$_0$-post$_3$: n=18, post$_3$-post$_6$: n=42), therefore p-values should be interpreted with caution.
