# Supplementary material for: Preventive effect of sensorimotor exercise and resistance training on chemotherapy-induced peripheral neuropathy: a randomised-controlled trial
Source: Br J Cancer. 2021 Jul 5;125(7):955–65. doi: 10.1038/s41416-021-01471-1 (PMC8476560; doi:10.1038/s41416-021-01471-1)
Supplement: Supplementary file 10 — Table S8. Information on chemotherapy dose modifications, early termination and concomitant CIPN treatment and prevention measures. [file 41416_2021_1471_MOESM10_ESM.pdf]

Table S8. Information on chemotherapy dose modifications, early termination and concomitant CIPN treatment and prevention measures (based on the ITT study sample).

	Total	SMT	RT	UC	*p value*
**CHT dose modifications**	49 (32%)	12 (26%)	22 (40%)	15 (28%)	.239
- neuropathy	24 (15%)	8 (16%)	9 (15%)	7 (13%)	.917
- hematopoietic disturbances	8 (5%)	2 (4%)	5 (8%)	1 (2%)	
- skin reactions	5 (3%)		2 (3%)	3 (6%)	
- obstipation/diarrhea	5 (3%)		4 (7%)	1 (2%)	
- mucositis	4 (2%)		4 (7%)		
- chemotherapy intolerance symptoms	3 (2%)	1 (2%)		2 (4%)	
- arthralgia/myalgia	2 (1%)		1 (2%)	1 (2%)	
- cardiac signs or symptoms	1 (1%)		1 (2%)		
- thrombopenia	1 (1%)	1 (2%)			
- edema	1 (1%)	1 (2%)			
- liver reactions	1 (1%)			1 (2%)	
- nausea/vomiting	1 (1%)		1 (2%)		
- difficulty swallowing	1 (1%)	1 (2%)			
- psychological strain	1 (1%)		1 (2%)		
- reduced overall condition	1 (1%)		1 (2%)		
- extravasate	1 (1%)		1 (2%)		
- patient's wish	1 (1%)			1 (2%)	
- multiple reasons	3 (2%)	2 (4%)		1 (2%)	
- not known	2 (1%)		1 (2%)	1 (2%)	
**CHT early termination**	33 (21%)	10 (21%)	11 (20%)	12 (22%)	.960
- neuropathy	9 (6%)	4 (9%)	2 (4%)	3 (6%)	.573
- patient's wish	4 (3%)	1 (2%)	2 (4%)	1 (2%)	
- skin reactions	2 (1%)	1 (2%)	1 (2%)		
- hematopoietic disturbances	1 (1%)			1 (2%)	
- cardiac signs or symptoms	1 (1%)			1 (2%)	
- thrombopenia	1 (1%)			1 (2%)	
- haemoptysis	1 (1%)			1 (2%)	
- liver reactions	1 (1%)			1 (2%)	
- mucositis	1 (1%)		1 (2%)		
- obstipation/diarrhea	1 (1%)		1 (2%)		
- arthralgia/myalgia	1 (1%)			1 (2%)	
- progressive disease	1 (1%)	1 (2%)			
- multiple reasons	5 (3%)	3 (6%)	2 (4%)		
- not known	4 (3%)		2 (4%)	2 (4%)	
**Concomitant CIPN treatment / prevention measures**					
- CIPN specific medication	16 (10%)	6 (12%)	5 (9%)	5 (9%)	.792
- pain oil	5 (3%)	1 (2%)	2 (4%)	2 (4%)	
- painkillers	2 (1%)	1 (2%)	1 (2%)		
- Vitamin B/D	103 (63%)	32 (65%)	39 (68%)	32 (56%)	.371
- food supplements	27 (17%)	10 (20%)	12 (21%)	5 (9%)	
- ice	66 (40%)	19 (39%)	23 (40%)	24 (42%)	.941
- massages	56 (34%)	15 (31%)	19 (33%)	22 (39%)	.675
- general mobility exercises	14 (9%)	4 (8%)	5 (9%)	5 (9%)	
- (Kneipp) bathing	7 (4%)	2 (4%)	4 (7%)	1 (2%)	
- handworks/handcrafts	7 (4%)	1 (2%)	3 (5%)	3 (5%)	
- acupuncture	4 (2%)	2 (4%)	2 (4%)		
- electrostimulation/TENS	1 (1%)		1 (2%)		
- vibration	1 (1%)		1 (2%)		
- yoga	1 (1%)		1 (2%)		
- others	6 (4%)		6 (11%)		
